# Long-Term Results of Femorodistal Sequential Composite-Bypass Combining Heparin-Bonded PTFE-Prosthesis and Autologous Vein Using the Deutsch Bridge Technique in Critical Limb-Threatening Ischemia

**DOI:** 10.3390/jcm12082895

**Published:** 2023-04-16

**Authors:** Achim Neufang, Valerian Zhghenti, Carolina Vargas-Gomez, Thomas Umscheid, Peter von Flotow, Rainer Schmiedel, Savvas Savvidis

**Affiliations:** 1 Department of Cardiac and Vascular Surgery, University Medical Center, Johannes Gutenberg-University, 55131 Mainz, Germany; 2 Department of Vascular Medicine, Helios Dr. Horst Schmidt Hospital (Teaching Hospital of Johannes Gutenberg-University, Mainz), 65199 Wiesbaden, Germany; 3 Vascular Surgery, University Hospital Son Espases, 07120 Palma de Mallorca, Spain; 4 Department of Vascular Medicine, Westpfalz-Klinikum II Kusel (Teaching Hospital of Johannes Gutenberg-University School of Medicine), 66869 Kusel, Germany

**Keywords:** limb salvage, blood vessel prosthesis, heparin-bonded graft, human, vascular patency

## Abstract

Background: Autologous vein bypass provides excellent long-term results in critical limb-threatening ischemia (CLTI), but a substantial portion of patients have insufficient vein length. In limbs with two distal outflow vessels and limited vein length, a vascular prosthesis may be combined with autologous vein for a sequential composite bridge bypass (SCBB). Results regarding graft function, limb salvage and reinterventions are presented. Methods: Between January 2010 and December 2019, 47 consecutive SCBB operations with a heparin-bonded PTFE-prosthesis and autologous vein were performed. Grafts were followed with a duplex scan with prospective documentation in a computerized vascular database. Retrospective analysis of graft patency, limb salvage and patient survival was performed. Results: Mean follow-up was 34 months (range 1–127 months). 30-day mortality was 10.6% and 5-year patient survival 32%. Postoperative bypass occlusion occurred in 6.4% and late occlusions or graft stenoses in 30%. Two prostheses developed late infection and seven legs were amputated. Primary, primary assisted, secondary patency and limb salvage rate were 54%, 63%, 66% and 85% after 5 years, respectively. Conclusions: SCBB patency and limb salvage were good despite a high early postoperative mortality. Combination of a heparin-bonded PTFE-prosthesis and autologous vein appears to be a valuable tool in CLTI in case of insufficient vein.

## 1. Introduction

In CLTI tibial or pedal bypass surgery with autologous vein provides excellent long-term graft patency and limb salvage. The use of a single-segment greater saphenous vein is recommended as first-line treatment [1]. Despite advances in endovascular therapy, bypass surgery must be considered after failed endovascular recanalization attempts or reocclusion with recurrence of CLTI. On the other hand, reoperation may become necessary after the failure of a previous bypass. For all peripheral bypasses, reconstruction with autologous vein is recommended because of the significantly better patency and limb salvage compared to prosthetic bypass. Unfortunately, there remains a substantial portion of patients who lack of sufficient vein length. In this situation, synthetic or biologic vascular prostheses must be considered as graft material. The outcome of prosthetic femoro-distal bypass remains inferior compared to autologous vein in terms of patency and limb salvage, although vein patches or cuffs for specific anastomotic configurations yield improved results [2,3,4]. For many years the use of the sequential anastomotic composite technique in the case of two possible outflow vessels has been available to improve graft outflow into the distal arterial bed, combining available good-quality vein segments with a synthetic or biological prosthetic inflow graft [5,6,7,8,9,10,11]. Historic results of standard PTFE-prosthesis with a direct arterial anastomosis have been moderate in the treatment of femoropopliteal disease and even worse in femorotibial bypass [12]. More recent studies with heparin-bonded PTFE-prostheses showed improved results with respect to limb salvage and bypass patency [13,14,15]. Deutsch introduced the SCBB as a promising sequential bypass technique combining a distal vein bridge with a standard PTFE-prosthesis inflow bypass to improve patency [16]. The results of a single-center consecutive series of SCBB combining autologous vein with a heparin-bonded PTFE-prosthesis are analyzed.

## 2. Materials and Methods

All SCBBs with a heparin-bonded PTFE-prosthesis performed between January 2010 and December 2019 were identified out of 1663 infrainguinal bypasses documented in a computerized vascular database. In the case of a patient having an autologous vein with an insufficient length and the identification of two patent distal arteries the SCBB technique was chosen after evaluation of endovascular options. Follow-up data were entered prospectively in a computerized vascular database according to a follow-up scheme. Perioperative data with patient characteristics and follow-up data were evaluated retrospectively.

Operative technique and graft configuration: Generally, an all-autogenous reconstruction with ipsi- or contralateral greater saphenous vein, arm veins or lesser saphenous vein including spliced vein grafts was intended whenever possible. Only if the harvested suitable vein or spliced vein segments were insufficient to reach the angiographically visible artery considered to be the best potential outflow vessel with pedal run-off was the SCBB technique chosen. Vein segments of adequate caliber were combined with a heparin-bonded PTFE-prosthesis (Propaten^®^) as the proximal part of the construction. To enhance bypass outflow, a patent popliteal or tibial artery segment in addition to the best visible outflow artery was integrated into the reconstruction resulting in distal sequential anastomoses. Bypass configuration was chosen according to the available vein length and the site of the recipient vessels. The distal arteries were interconnected with the autologous vein in an end-to-side technique according to the bridge technique proposed by Deutsch [16]. If necessary, this vein bridge was constructed with good quality vein segments that were spliced together in order to connect both distal outflow vessels with autologous vein. Destruction of the vein valves with a modified Leather retrograde valvulotome (V. Mueller Care Fusion, San Diego CA, USA) was performed to enable antegrade and retrograde blood flow in the vein bridge graft. 

**Figure 1:**
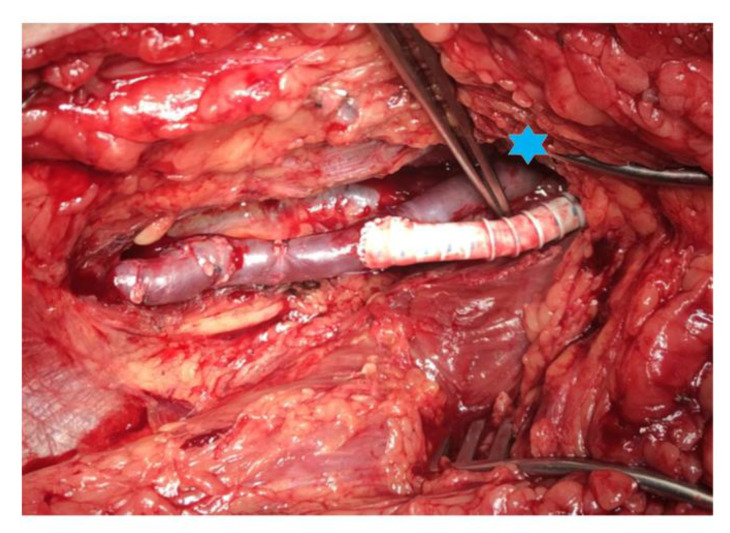
Vein bridge connecting the distal popliteal artery (*) and the distal posterior tibial artery with spliced greater saphenous vein. The heparin-bonded PTFE-prosthesis is anastomosed to the vein in end-to-side technique.

The heparin-bonded PTFE-prosthesis was prepared according to manufacturers’ instructions for use. After routine intravenous administration of 5000 IU heparin the proximal anastomosis was performed preferentially at the common femoral artery with additional endarterectomy or profundaplasty if necessary. The PTFE-prosthesis was placed in a subfascial layer whenever possible. Construction of the venous part was performed according to the anatomic relation of the distal vessels and the vein graft placed either in a partial subcutaneous or subfascial position. The anastomosis of the PTFE-prosthesis was placed in end-to-side technique at the proximal part of the vein bridge (Figure 1). Distal anastomoses were constructed with polypropylene suture (7.0) in a conventional manner. 

Transit time flow measurement (Cardio-Med Medi-Stim, Oslo, Norway) in the venous part of the construction assessing flow in both directions of the vein bridge whenever possible was used to confirm graft patency intraoperatively. Prior to discharge patency and graft configuration were documented by means of duplex scan and/or angiography (MRA or CTA). Minor amputations were performed if necessary after demarcation of the necrotic tissue. Heparin was provided intravenously in combination with additional antiplatelet medication postoperatively. For long-term antithrombotic therapy oral anticoagulation with phenprocoumon (Marcumar^®^) was preferred and was started within a few days postoperatively with an intended INR level of 2.5. Sole long-term antiplatelet medication was preferred in case of contraindications for oral anticoagulation, or during the later course when bleeding complications or poor patient compliance mandated a change in the anticoagulation regimen. 

An indefinite follow-up scheme with outpatient clinic visits and duplex ultrasound examination of the bypass graft after 3, 6, 12, 18 and 24 months with annual repetitions thereafter was started. Graft patency was classified according to the recommended standards for reports dealing with lower extremity ischemia [17]. For statistical analysis, preservation of the distal venous part or a patent vein bridge in case of occlusion of the prosthetic graft, complete prosthetic graft replacement or major amputation with a patent bypass graft was regarded as bypass failure. Data were prospectively documented in a computerized database (ACCESS 2000 for windows). Retrospective analysis was performed with Kaplan–Meyer survival test and log rank test (SPSS 28.0 for windows) Documentation and analysis followed the standards for clinical studies defined by the ethics committee of the state of Rhineland–Palatinate, Germany.

## 3. Results

### 3.1. Epidemiological Data and Indication for Operation

Over a 9-year period, 47 consecutive SCBBs with a vein bridge and heparin-bonded PTFE were constructed in 46 patients with a mean age of 79.2 ± 10.1 in 47 lower limbs. This represents 2.8% of bypass operations during the reported time period. A total of 48% of the patients were older than 80 years. All operations were performed for CLTI. One patient with end-stage renal disease underwent staged bilateral operations. In 11 limbs (23%), the SCBB was the first ipsilateral revascularization procedure. In all other cases, various vascular operations and interventions had been performed. Mean operative time was 270 (±81; 166–491) minutes. Patient demographics, risk factors, comorbidities, previous procedures and indications for surgery are listed in Table 1 and Table 2. Data from selected blood tests, antithrombotic and statin medication are listed in Table 3.

### 3.2. Bypass Configuration

**Figure 2:**
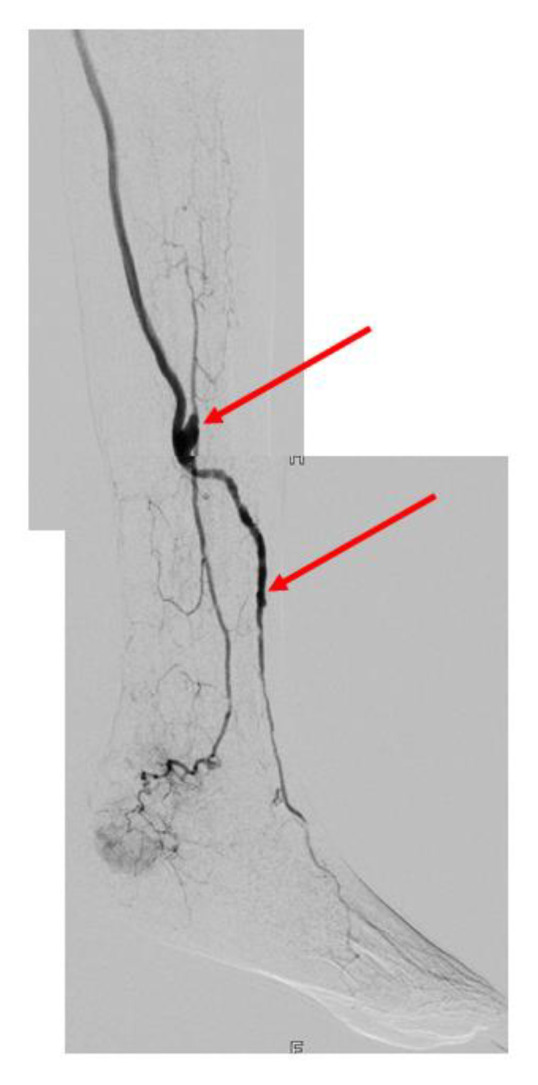
Basilic vein bridge between peroneal and anterior tibial artery (arrows) with proximal heparin-bonded PTFE-prosthesis. Image from 84 months postop.

Heparin-bonded PTFE-prostheses with a diameter of 6 mm were chosen for the proximal part of the bypass except in one case where an 8 mm graft was used. The vein bridge consisted of an arm vein alone (Figure 2) in 62% and of the greater saphenous vein in 25% of cases. In 5 cases, arm vein and saphenous vein were combined and in one case the vein bridge was constructed with the contralateral posterior tibial vein. In 30%, vein segments were spliced together to achieve the necessary length for the planned vein bridge configuration. All proximal anastomoses were located at the groin level. The composite anastomosis was placed in the distal part of the calf in 60% and the most distal bridge anastomosis was placed at the lower calf or the foot level in 58%. Details regarding bypass material, configurations and locations of the anastomoses are listed in Table 4 and Table 5.

### 3.3. Patient-Related Outcome

**Figure 3:**
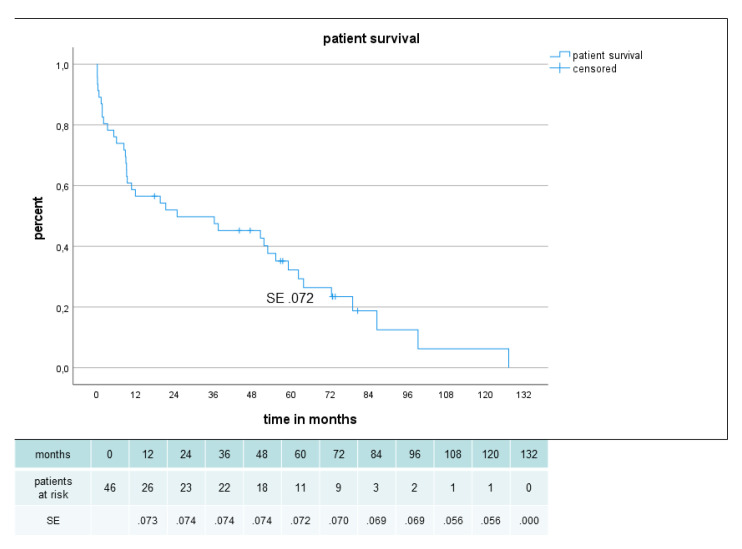
Patient survival in 46 patients with sequential composite bridge bypasses with a heparin-bonded PTFE-prosthesis and autologous vein.

Thirty-day mortality was 10.6% (5 patients). One patient refused necessary hemodialysis, the other patients died from cardiac failure, visceral ischemia and pneumonia. Early postoperative complications are listed in Table 6. Lifelong follow-up information with a mean follow-up of 34.5 ± 32.8 months (range 1–127 months) was available in all patients except one patient who was lost after major amputation. A total of 18 patients could be followed for more than 48 months, and 38 patients died during follow-up mostly from cardiovascular events or malignant tumors. Two patients died after major amputation after 6 and 2 months, respectively. There was no mortality after other secondary vascular procedures. In the 38 patients who died during follow-up, the bypass was patent in 30 cases and the limb was viable in 33 cases. Overall patient survival was 52% at 2 years and 32% at 5 years postoperatively (Figure 3). There was no significant gender-related difference for late survival (p .227).

### 3.4. Graft Function and Limb Salvage

There were two complete early bypass occlusions. In one case, this was corrected by means of thrombectomy but the bypass reoccluded at 15 months, necessitating a new bypass with durable limb salvage. In the second case, above-knee amputation became necessary after one month. One asymptomatic partial bypass occlusion of the distal part of the vein bridge was treated conservatively. The bypass remained patent.

**Figure 4:**
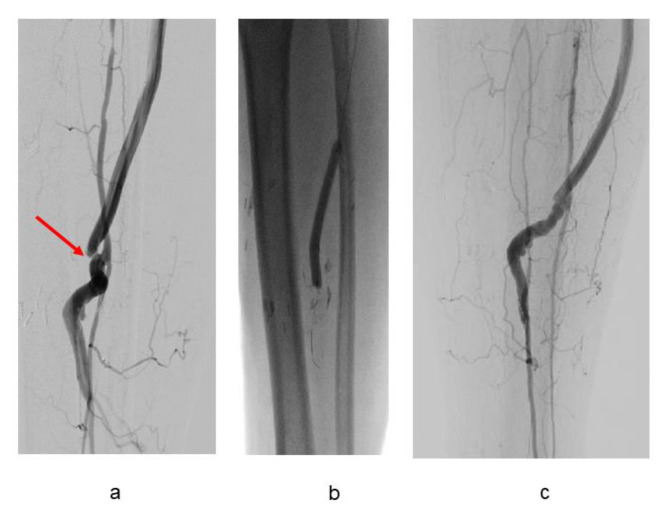
(**a**–**c**). High-grade stenosis of composite stenosis (arrow) (**a**) detected 23 months postop. Successful angioplasty with drug-eluting balloon (**b**,**c**).

A total 38 grafts (83%) could be followed with repetitive duplex investigations. Duplex scans detected significant stenotic lesions (Figure 4a) in five (10.6%) patent grafts. To maintain bypass patency, five additional interventions with drug--eluting balloon angioplasty (Figure 4b,c) including thrombolysis became necessary. These interventions resulted in durable bypass function in four cases. Nine bypasses (19.1%) failed during follow-up. Successful reoperation with a new prosthetic graft was possible in two instances with durable limb salvage. Two grafts developed late infection with bypass occlusion after 6 and 55 months, respectively, resulting in graft removal in both cases with major amputation in one case and limb preservation by local femoral artery reconstruction in the second case. Severe ischemia after bypass occlusion resulted in major amputation in three patients and conservative treatment was possible in two patients. Graft failure, revision techniques and other procedures are outlined in Table 6 and Table 7.

**Figure 5:**
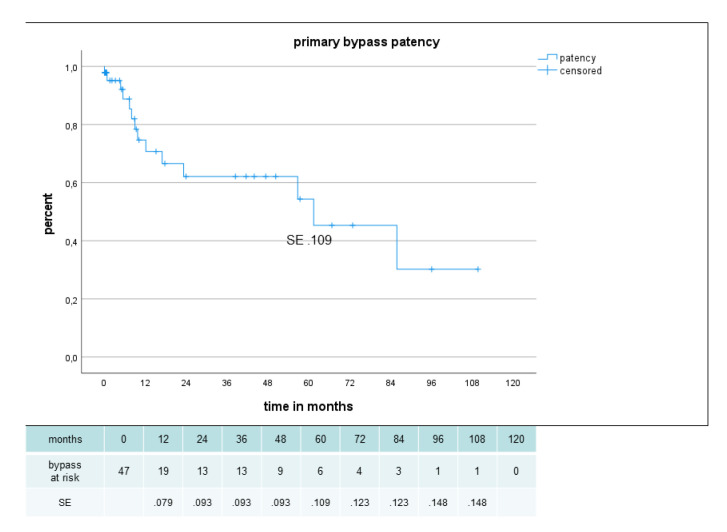
Primary patency in 47 sequential composite bridge bypasses with a heparin-bonded PTFE-prosthesis and autologous vein.

**Figure 6:**
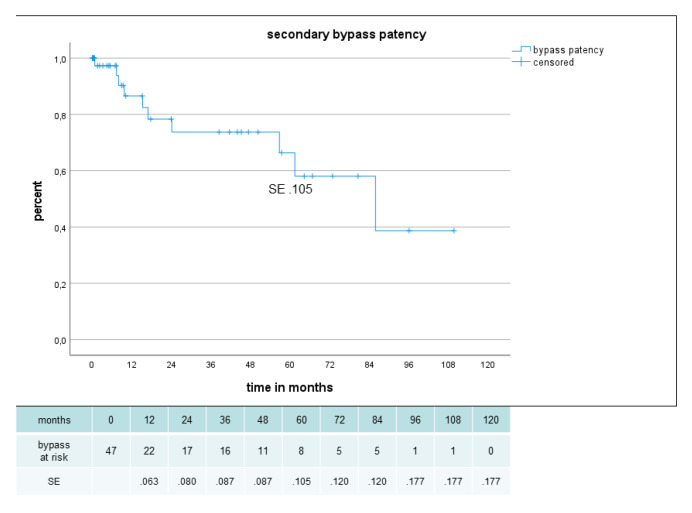
Secondary patency in 47 sequential composite bridge bypasses with a heparin-bonded PTFE-prosthesis and autologous vein.

**Figure 7:**
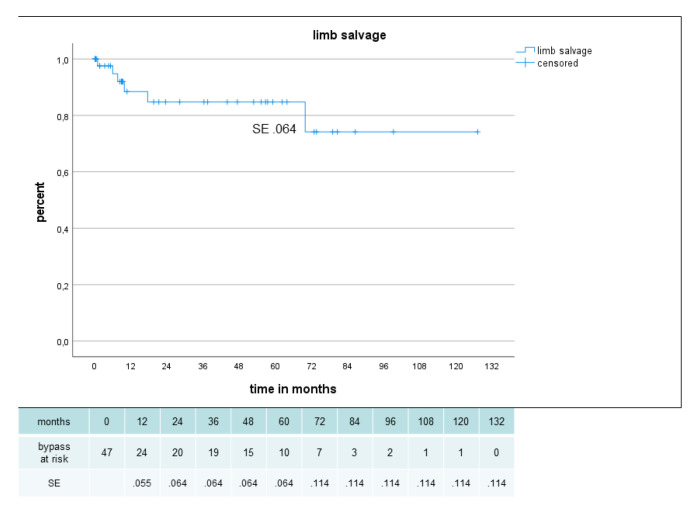
Limb salvage in 47 sequential composite bridge bypasses with a heparin-bonded PTFE-prosthesis and autologous vein.

**Figure 8:**
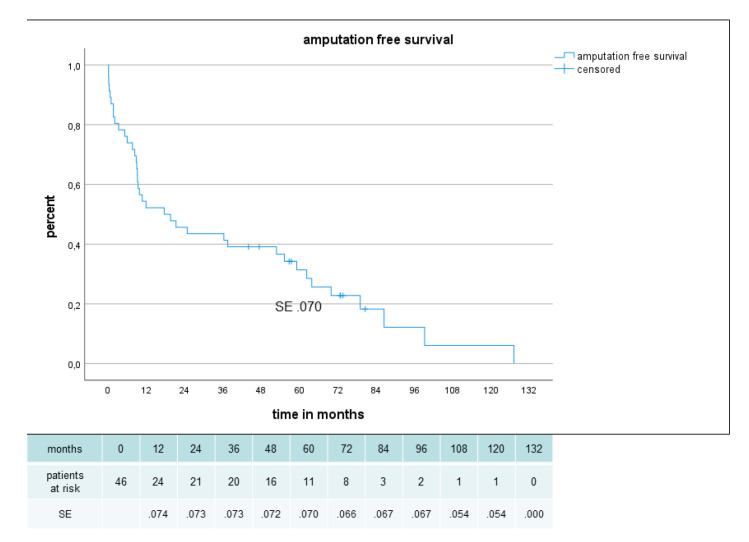
Amputation free survival in 46 patients with sequential composite bridge bypasses with a heparin-bonded PTFE-prosthesis and autologous vein.

Primary, primary assisted, secondary patency and limb salvage rate were 62%, 80%, 80% and 85%, respectively, after 2 years and 54%, 63%, 66% and 85%, respectively, after 5 years (Figure 5, Figure 6 and Figure 7). There were no significant difference in secondary patency for diabetes (p .556) or gender (p .698) and in limb salvage for diabetes (p .264) or gender (p .778). Amputation-free survival was 45% after 2 years and 31% after 5 years with no significant difference for diabetes (p .648) or gender (p .180) (Figure 8).

## 4. Discussion

Autologous vein should be used for all infrainguinal bypass reconstructions whenever possible due to the superior results achieved compared to prosthetic material [18,19]. Recent data has given evidence for superior results for greater saphenous vein bypass compared to endovascular treatment for CLTI [1]. Despite all efforts, there is still a substantial need for prosthetic bypass in case of insufficient vein length. Unfortunately, prosthetic bypass grafting onto tibial and pedal arteries yields significantly inferior results compared to autologous reconstructions despite the use of special anastomotic techniques including vein patches or cuffs [15]. One concept to improve long-term bypass graft patency is based upon the reduction of outflow resistance for the bypass in the peripheral vascular tree by including more than one possible outflow vessel into the reconstruction. This concept of sequential anastomoses was introduced more than 30 years ago. Additionally to the increased flow rate patency of the bypass may be preserved by the more proximal anastomosis in the case of late occlusion of the more distal part of the reconstruction without severe recurrent ischemia [7].

The most frequently described sequential bypass technique was the jump graft technique with prosthetic anastomosis at the popliteal artery combined with a distal extension with autologous vein [20,21,22,23]. If the venous part of the bypass originates from the proximal prosthesis, primary patency rates between 35 and 64% with a limb salvage rate of 64 to 84% between two and five years after operation were reported; however, the risk of occlusion of the venous part was reported in the case of prosthetic inflow occlusion. A number of historical series outlined satisfactory patency and limb salvage rates for the sequential composite bypass technique. Very satisfactory results were found in more recent series with good bypass patency and 5-year limb salvage rates up to 87% (Table 8) [8,11,16,20,21,22,23,24,25,26,27,28,29]. 

One distinct advantage reported for sequential bypass configurations is the fact that if failure of the prosthetic bypass part occurs, the distal sequential autologous part may remain patent and recurrent ischemia may be only moderate. In selected cases this preserved venous part may be used for a redo procedure. 

Deutsch introduced a promising and simple composite technique that avoids contact between the PTFE-prosthesis and the distal arteries by connecting both outflow vessels with a distal vein bridge. A proximal PTFE-prosthesis is then anastomosed to this vein bridge [16]. A distinct advantage of his technique is that all anastomoses are performed in end-to-side technique and the length of the bridge may be adapted to the suitable anastomotic site of the distal arteries and the length of the available good quality vein material. Gargiulo reported a favorable five-year primary patency rate of 65% and a four-year limb salvage rate of 85% with a modification of this technique [26]. Our own group also reported promising results with this technique using a biological vascular prosthesis for inflow graft. The bridge graft was used in 40% of cases [28].

All historical reported series were performed with standard PTFE-protheses or biological grafts. The heparin-bonded PTFE graft has evolved into a new alternative to the commonly used synthetic prostheses. It is especially remarkable that the reported early results for below-knee bypasses in critical ischemia were favorable [14]. However, the results with this prosthesis remain inferior in comparison to autologous vein grafts [30,31,32]. Until today, no published data exist that analyze the patency of the heparin-bonded prosthesis in complex sequential reconstructions. Our report is the first to analyze the results with this prosthesis. We found a promising secondary patency rate of 66% after five years with an excellent limb salvage rate of 85% in a series where the level of the most distal anastomosis was at the ankle or below in 58% of cases. It must be emphasized that in our reported series the rate of re-do bypasses (64%) was very high. Only in 23% of patients was the peripheral vascular system untouched. As in other reports, we found that in the later course the risk for major amputation was high in cases of complete bypass failure and impossible further revascularization. For this reason, we believe that long-term duplex scan surveillance may detect those stenotic lesions that threaten graft patency. It should be used as long as the patient may have benefit from a possible prophylactic intervention on the bypass to preserve a functional limb for independent ambulation. An individualized approach with preference of endovascular methods helped to avoid graft failure in most cases with detected bypass stenoses. For this reason, we perform duplex scan surveillance for complex reconstructions for the rest of the patient’s life, or at least as long as the patient is able to ambulate.

There are several limitations in the interpretation of our study. It is a single-center retrospective study on a small number of patients that represents only a small percentage of CLTI patients treated. The rate of redo revascularisations was above 60% and the mean patient age was 79 years. A total of 48% were older than 80 years. This does not allow comparison with larger studies focusing on autologous vein or popliteal prosthetic bypass only for CLTI. Our reported early mortality with 10.6% was high, even taking into account that in one case the patient had rejected dialysis; it was significantly above the mortality rate of 5% accepted in the international guidelines. Only 52% of our patients were alive after 2 years. A reason for this poor patient prognosis may be seen in the significantly reduced cardiovascular and renal status at the time of operation. On the other hand, the limb salvage rate of this technique was almost similar to that with vein and even very old patients remained mobile due to the preserved limb. 

This may reflect the situation of an emerging number of old and very old high-risk patients with CLTI with the need for surgical revascularization despite endovascular techniques. They definitely represent a difficult subset of vascular patients. In none of the patients in our reported series did a viable endovascular option exist after discussion of the case with the vascular interventionalist. A less-invasive surgical approach with a hybrid technique would have been possible only in a few patients. Only primary amputation would have been a time-saving alternative. However, even in a modern series for primary major amputation in CLTI patients, 30 day mortality was reported to be between 10.8% for the lower-risk score and 28.7% for the higher-risk score patients. [33] With respect to the poor prognosis for primary amputation, a surgical revascularization approach may be discussed in this high-surgical-risk group with no good endovascular option to preserve patient mobility with a functional limb besides a strict conservative management. The SCBB developed by Deutsch is associated with good late bypass patency and an excellent limb salvage rate if performed with a heparin-bonded PTFE-prosthesis. It appears to be a valuable tool in those situations where adequate vein length is missing and prosthetic bypass outflow may be enhanced by inclusion of more than one vessel into the arterial reconstruction. 

## 5. Conclusions

The reported patency and limb salvage rates for complex SCBB with the Deutsch bridge technique and a heparin-bonded PTFE-prosthesis support the further use of this technique in appropriate CLTI cases with limited vein material. Lifelong duplex scan surveillance combined with patency-maintaining interventions seems to be effective and leads to a very favorable secondary patency and limb salvage. Although this technique is clearly more invasive than a single-outflow prosthetic bypass, the additional effort may be rewarded by enhanced patency and limb salvage. Further studies with this technique in patients with unsuitable veins are necessary. 

## Figures and Tables

**Table 1:** Patient data and previous procedures.

	Patients n = 46	%
Mean age ± SD range (y)	79.2 ± 10.1 54.1–93.7	
Age over 80 y	22	48
female	23	50
male	23	50
**Risk factors and comorbidities**	**n**	**%**
Hypertension	46	100
Hyperlipidemia	36	78.2
Diabetes mellitus	20	43.5
Nicotine abuse	13	28.2
Cardiac status *		
Category 0	3	6.5
Category 1	28	60.8
Category 2	8	17.5
Category 3	7	15.2
Coronary artery disease	34	73.9
Previous cerebrovascular accident	16	34.7
Renal status		
Creatinine < 1.5 mg/dL	29	63.1
Creatinine > 1.5 and <2.4 mg/dL	10	21.7
Creatinine > 2.4 and <6.0 mg/dL	2	4.3
dialysis	5	10.9
**Previous ipsilateral vascular procedures in 47 limbs**	**n**	**%**
No previous ipsilateral vascular intervention or operation	11	23.4
Infrainguinal bypass **	28	63.8
Local reconstruction of femoral artery ***	10	21.3
Endovascular procedure	18	38.2
Infrainguinal ipsilateral stent	5	10.6
Surgical and endovascular procedure	8	17.0
Surgical inflow procedure	5	10.6
Endovascular inflow procedure	4	8.5

* according to reporting standards [17]. ** one or two failed previous bypass procedures. *** eight in combination with previous bypass.

**Table 2:** Indications for composite sequential bypass surgery.

Indication	n	%
critical limb threatening ischemia	47	100
rest pain	8	20.5
necrosis/ulceration	38	75.5
acute ischemia on severe chronic ischemia *	1	2.4
total	47	100

* acute thrombotic occlusion of severely atherosclerotic diseased superficial femoral artery in combination with chronic popliteal and tibial occlusions.

**Table 3:** Blood tests, antithrombotic and statin medication at admission and 30 days.

Blood Test Preop	Value (Range)	
Hemoglobin mg/dL	11.8 ± 1.8	
Thrombocytes per microliter	261.000 ± 105.000	
Leukocytes per microliter	9.5 ± 3.4	
CRP	3.5 ± 5.0	
Creatinine mg/dL	1.6 ± 1.4 (0.5–6.8)	
GFR mL/min	56.5 ± 34.0	
Medication preop	N	(%)
Aspirin	35	76.1
Clopidogrel	4	8.6
Vit K antagonist	15	32.6
DAOC	1	2.2
Statin	32	69.5
Medication postop		
Aspirin	42	91.3
Clopidogrel	12	26.1
Vit K antagonist	23	50.0
DAOC	2	4.4
Statin	39	84.7

CRP (c-reactive protein), GFR (Glomerular filtration rate); DAOC (direct oral anticoagulation).

**Table 4:** Bypass graft material and configuration.

Prosthetic Part	n	%
Heparin-bonded PTFE-prosthesis	47	100
diameter 6 mm	46	98
diameter 8 mm	1	2
venous (bridge) part *		
greater saphenous vein	12	25
arm vein	29	62
Combination of greater saphenous vein and arm vein	5	11
posterior tibial vein	1	2
total	47	100

* In 14 bypasses, up to three vein segments had to be spliced together to achieve the necessary length for the venous bridge. A total of 62 vein segments were used in 47 composite bypasses.

**Table 5:** Location of anastomoses.

Proximal Anastomosis	n	%
Common femoral	40	85
Profunda femoris	4	9
Inflow bypass	2	4
External iliac artery	1	2
**Composite anastomosis**		
Distal thigh	1	2
Proximal calf	18	38
Distal calf	28	60
**First sequential anastomosis**		
Popliteal artery (below knee in 9 cases)	10	21
Proximal part of tibial artery	6	13
Middle and distal part of tibial artery	31	66
**Second sequential anastomosis**		
Proximal and middle part of tibial artery	20	42
distal part of tibial artery *	5	11
pedal artery *	22	47

* 58% of the second distal anastomoses at the level of the ankle or below.

**Table 6:** Early (30-day) patient-related and bypass-related complications.

Postoperative (30 d or in Hospital) Complications in 47 Operations	n	%
death (ischemic heart failure, pneumonia, visceral ischemia, renal failure *	5	10.6
Myocardial infarction, cardiac failure, arrhythmia	7	14.8
stroke, TIA	1	2.1
respiratory failure, pneumonia	3	6.4
Visceral artery occlusion	1	2.1
Other Arterial occlusion	1	2.1
renal failure (reversible) *	3 (2)	6.4 (4.2)
sepsis, systemic inflammatory signs	3	6.4
delirium	3	6.4
others	1	2.1
Bypass occlusion complete **	2	4.2
Occlusion of second distal anastomosis (asymptomatic, left untreated)	1	2.1
hematoma (surgical revision)	0	0
Superficial wound infection/delayed wound healing	6	12.7
Prosthetic graft infection	0	0
combined mortality and morbidity ***	22	46.8
Major amputation	1	2.1
Minor amputation	10	21.3

* One patient refused dialysis and died from uremia; ** one bypass revision with successful thrombectomy, one above-knee amputation; *** 34 local or systemic complications in 22 operations.

**Table 7:** Late patient and bypass outcome.

Late Patient Outcome Mean Follow-Up 34.5 ± 32.8 mo (1–127 mo)	n	%
Death during follow-up (cardiovascular, tumor) including early mortality	38	82.6
5 y patient survival (Kaplan–Meier)		32%
**Late bypass and limb outcome**		
Bypass or anastomotic stenosis	5	11
PTA proximal anastomosis	1	2
PTA distal anastomosis	2	4
PTA Bypass	2	4
Bypass occlusion	11	23
New bypass	2	4
Successful lysis	1	2
Major amputation	5	11
Bypass explantation (infection) *	2	4
Prosthetic graft infection **	2	4
Major amputation (below knee) ***	6 (4)	13

* With bk amputation in one case; ** both infections combined with late acute bypass occlusion; *** one late major amputation despite patent bypass.

**Table 8:** Historical and actual data for sequential composite bypass with vascular prostheses.

Author	Year	nr	Prosthesis	Configuration	Popliteal Artery	At Ankle Pedal	Re-Do	Prim. Pat. %	Sec. Pat. %	Limb Salv %
Verta [11]	1982	54	PTFE	Jump graft	yes	yes	unclear	Not given	72.4 (4y)	78 (4y)
Flinn [8]	1984	30	PTFE	Jump graft	yes	unclear	unclear	Not given	80 (2y)	?
McCarthy [21]	1992	67	PTFE	Jump graft	yes	yes	>50%	40 (4y)	Not given	70 (4y)
Chang [25]	1995	55	PTFE	Serial anastomoses	yes		75%	35 (8y)	52 (8y)	65 (8J)
Alexander [20]	1996	35	PTFE	unclear	yes	unclear	unclear	35 (2y)		60 (2y)
Bastounis [24]	1999	21	PTFE	Serial anastomoses	no	unclear	no	59 (5y)	unclear	80 (5J)
Oppat [22]	1999	102	PTFE	Jump graft	yes	unclear	unclear	20 (7y)	unclear	Similar to patency
Deutsch [16]	2001	45	PTFE	Vein bridge	no	yes	62%	26 (4y)	39 (4y)	45 (4y)
Roddy [23]	2002	27	PTFE	Arterial origin of distal vein graft	yes	yes	>90%	64 (3y)	64 (3y)	88 (3y)
Mahmood [27]	2002	68	PTFE	Side by side anastomosis	yes	yes	49%	68 (2y)	73 (2y)	75 (2y)
Gargiulo [26]	2010	25	PTFE	Vein bridge	88%	unclear	64%	54 (5y)		85 (4y)
Neufang [28]	2014	122	HUV ovine collagen	Various configurations	43%	67%	30%	48% (5y)	71% (5y)	87% (5y)
Rogers [29]	2016	6	PTFE	Diamond-shaped anastomosis	6/6	unclear	unclear	6/6 Limited follow-up	6/6	5/6
Neufang	2023	47	PTFE heparin bonded	Vein bridge	21%	58%	63%	54% (5y)	66% (5y)	85% (5y)

## Data Availability

Analyzed data are available from the corresponding author. Primary data belong to the patients records and are unavailable to the public.
